# Repetitive compound muscle action potentials in electrophysiological diagnosis of congenital myasthenic syndromes: A case report and review of literature

**DOI:** 10.4103/0972-2327.64645

**Published:** 2010

**Authors:** R. Shiva Kumar, Abraham Kuruvilla

**Affiliations:** Department of Neurology, Sree Chitra Tirunal Institute for Medical Sciences and Technology, Trivandrum, Kerala, India

**Keywords:** Congenital myasthenic syndromes, slow-channel syndrome, repetitive CMAPs

## Abstract

Congenital myasthenic syndromes (CMSs) are a heterogeneous group of disorders, characterized by dysfunction of neuromuscular junction (NMJ) transmission. These syndromes are genetically inherited and are present since birth. Some have characteristic clinical or electrodiagnostic features but in many cases determination of the specific form requires genetic studies or specialized morphological and electrophysiological studies on muscle tissue. We report a case of a 4-year-old boy with progressive ptosis and limitation of ocular movements who was diagnosed as slow-channel CMS based on the characteristic electrodiagnostic features. Repetitive compound muscle action potentials (R-CMAPs) were recorded after single nerve stimulus, with decremental response after repetitive trains performed at 3 Hz. CMSs are at times clinically difficult to distinguish from acquired myasthenia. The characteristic clinical and electrodiagnostic features help in the diagnosis and enable rational therapy. In this article we discuss the characteristics of synaptic R-CMAPs.

## Introduction

Myasthenia gravis (MG) in infancy and childhood are of two types: congenital or acquired. Congenital myasthenic syndrome (CMS) includes a number of phenotypically and genetically heterogeneous disorders that are familial, with affected persons lacking autoantibodies against acetylcholine receptor (AChR).[1] Based upon clinical, ultrastructural, *in vitro* electrophysiological, and molecular genetic methods of study, these heterogeneous types of CMS have recently been tentatively classified.[2] However, in most of the centers where advanced investigations are not possible, the diagnosis of CMS depends on the clinical features and the characteristic findings in neurophysiological studies. We report a case where the presence of double CMAP (R-CMAP) helped in the diagnosis of definite CMS.

## Case Report

**Figure 1:**
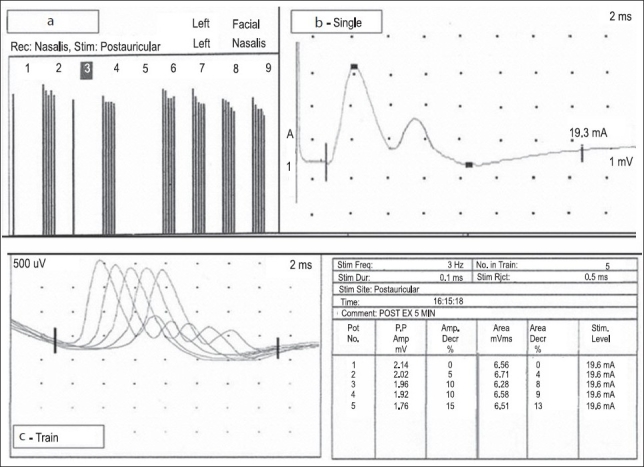
(a) Decremental response of 15% from the facial muscles. (b) Double CMAP was recorded after single stimulus from nasalis with the first CMAP larger than the second. (c) Shows appearance of post-exercise decremental response at 3 Hz in the second CMAP before the first CMAP

A 4-year-old-boy, born of nonconsanguineous parentage and with normal developmental milestones, presented with drooping of both eyelids of 1 year's duration. There was minimal diurnal fluctuation but there was history of worsening of the symptom on exertion. Neurological examination showed bilateral asymmetric ptosis and restriction of the extraocular muscles, with minimal signs of fatigability on exertion. No delayed pupillary light reflex was present. There was selective and severe weakness of the finger extensors. The deep tendon reflexes were reduced. The rest of the neurological examination was normal. There were no skeletal deformities and family history was negative. The neostigmine test was negative and serum AChR antibodies were not present. There was no evidence of thymic hyperplasia or thymoma. Repetitive nerve stimulation (RNS) test done from nasalis muscle at 3 Hz stimulation, showed a decremental response of abut 15% in both area and amplitude. Two CMAPs were recorded from the median, ulnar, and the facial nerves after a single stimulus [Figure 1a‐c]. The decremental response characteristically appeared first in the second CMAP rather than the first. No R-CMAP was recorded in the family members who were also tested. There was no response to cholinesterase medications. Based on the typical clinical features, the presence of R-CMAPs, and the decremental response on repetitive stimulation, we considered the diagnosis of postsynaptic congenital myasthenic syndrome, possibly slow-channel syndrome. The patient's symptoms are currently static. We plan to carry out a trial of quinidine, if required, during the follow-up.

## Discussion

CMSs are not uncommon but they are frequently misdiagnosed or go undiagnosed for a number of reasons. CMS can mimic several disorders and correct diagnosis requires specialized diagnostic methods that are available only at a few medical centers. The differentiation from autoimmune MG is important, as CMS does not respond to either thymectomy or immunosuppressive therapy. Two major features distinguish CMS from acquired MG, namely positive family history and absence of AChR antibodies. While a positive family history favors a diagnosis of CMS, a negative family history does not exclude it.[2]

The demonstration of R-CMAPs in completely resting muscle in the absence of a medication effect due to cholinesterase inhibitors is specific for diagnosis of CMS. Repetitive CMAPs in CMS are seen in two varieties, namely end-plate AChE deficiency (synaptic defect) and slow-channel syndrome (postsynaptic defect). The diagnostic clues for end-plate AChE deficiency are presence of R-CMAPs, refractoriness to cholinesterase medications, and delayed pupillary reflexes. In addition to the presence R-CMAPs, selective weakness of cervical muscles, wrist, and finger extensors and an autosomal dominant mode of inheritance favor the diagnosis of slow-channel CMS.

R-CMAPs seen after a single shock can be classified as being of either 'synaptic' or 'neural' origin. Synaptic R-CMAPs involve double (and rarely triple or more) discharges in disorders of the neuromuscular synapse, either because excess acetylcholine (ACh) remains present after the first discharge or because the normal amount of ACh causes reactivation in the slow-channel syndrome. Synaptic R-CMAPs may be initiated in the synapse, but they also depend on neural processes. The similarities between the 'double discharges' seen in partially denervated muscles (neural CMAPs) and in synaptic R-CMAPs make their distinction very difficult [Table 1]. The neural R-CMAPs occur in a group of diseases characterized by excess motor unit activity [i.e., continuous motor unit activity, Isaac syndrome, and neuromyotonia (NMT)].

**Table 1:** Characteristics of R-CMAPs[3]

	Synaptic R-CMAP	Neural R-CMAP
Origin	Abnormal synaptic event reactivates one muscle fiber, and can re-excite the nerve fiber, in turn exciting all muscle fibers of the same motor unit	Abnormal motor axon generates impulse trains, spontaneously or in the wake of a passing impulse; these activate the motor unit
Associated disorders	AChE blocking:	Neuromyotoni
	Drugs in MG	Familial paroxysmal kinesigenic ataxia and continuous myokymia
	Organophosphate poisons	
	CM with AChE deficiency	
	Slow-channel syndrome	
Number of extra discharges	One; rarely two	Up to 25

MG: myasthenia gravis; ACh: acetylcholine; AChE: acetylcholinesterase, CM: congenital myasthenia

In synaptic R-CMAPs seen after a single stimulus, characteristically, the amplitude of the first CMAP is larger than that of the second. On repetitive nerve stimulation, the decremental response occurs first in the second CMAP rather than in the first CMAP, as was seen in our case. Repeated stimulation diminishes the amount of available ACh; if the decline were to continue, the amount of ACh would change from an excess to a shortage. In electromyography (EMG) terms, the second component should disappear before the first component starts to show a decrement.[3] Latencies of 5–8 ms or 7–10 ms may occur between the first and second discharges.[4] In contrast, decrements of the first and second discharges may occur simultaneously. This can only be explained by differing abnormalities: that is, excess ACh causes repeated firing at some synapses, whereas a shortage causes others not to respond at all.

In clinical practice, the most common cause of R-CMAPs is cholinesterase medication. In its absence, and when repetitive discharges are present, CMSs can be reliably diagnosed in common practice. Our case illustrates the importance of electrophysiological testing in myasthenia: it aids early diagnosis of CMS and helps avoid unnecessary investigations and treatments like steroids, plasma exchange, and even thymectomy. In institutions like ours, where molecular diagnosis of CMS is not possible, the presence of synaptic R-CMAPs can aid in the diagnosis of CMS.

## References

[1] Engel A, Lambert E (1987). Congenital myasthenic syndromes. Electro Clin Neurophy.

[2] Engel A, Engel A, Franzini Armstrong (1984). In: Myasthenic syndromes. Myology basics and clinical.

[3] J Gert Van Dijk, Gert Jan Lammers, Axel R Wintzen (1998). Repetitive CMAPs: Mechanism of neural and synaptic genesis. Muscle and Nerve.

[4] Roth G (1980). Double discharges of distal origin: Influence on the firing rhythm. J Neural Sci.

